# Feasibility of using respiratory correlated mega voltage cone beam computed tomography to measure tumor motion

**DOI:** 10.1120/jacmp.v12i2.3473

**Published:** 2011-01-31

**Authors:** Mingqing Chen, R. Alfredo Siochi

**Affiliations:** ^1^ Department of Radiation Oncology University of Iowa Hospitals and Clinics Iowa City IA 52242 USA

**Keywords:** respiratory correlated, MVCBCT, tumor motion, non‐small‐cell lung cancer

## Abstract

The purpose of this study was to test the feasibility of using respiratory correlated mega voltage cone‐beam computed tomography (MVCBCT), taken during patient localization, to quantify the size and motion of lung tumors. An imaging phantom was constructed of a basswood frame embedded with six different‐sized spherical pieces of paraffin wax. The Quasar respiratory motion phantom was programmed to move the imaging phantom using typical respiratory motion. The moving imaging phantom was scanned using various MVCBCT imaging parameters, including two beam line types, two protocols with different ranges of rotation and different imaging doses. A static phantom was also imaged as a control. For all the 3D volumetric images, the contours of the six spherical inserts were measured manually. Compared with the nominal sphere diameter, the average relative error in the size of the respiratory correlated MVCBCT spheres ranged from 5.3% to 12.6% for the four largest spheres, ranging in size from 3.6 cc to 29 cc. Larger errors were recorded for the two smallest inserts. The average relative error in motion was 5.1% smaller than the programmed amplitude of 3.0 cm. We are able to conclude that it is feasible to use respiratory correlated MVCBCT to quantify tumor motion for lung cancer patients.

PACS numbers: 87.19.Wx, 87.57.Q

## I. INTRODUCTION

Lung tumor motion is of great concern in radiotherapy. Among the respiratory compensation techniques presently used, respiratory gating based on external surrogates has the advantage of requiring no extra fluoroscopy imaging dose^(1)^ or surgery to implant fiducials.^(^ ^2^ ^,^ ^3^ ^)^ Our clinic uses a strain gauge to record respiratory phases and normalized amplitudes for 4DCT imaging. We analyze these images to determine the strain gauge phases to use for gating the treatment beam and limit the treated motion to less than 1 cm. However, since tumor motion may change, the gating phases determined from the 4DCT may not represent the same displacement at the time of treatment. Large residual tumor motion with a high target miss rate (of about 30%) has been reported when using abdominal surface motion for the gating signal.^(^ ^4^ ^,^ ^5^ ^)^ The imprecision of using the abdominal surrogate alone can cause tumor underdose or normal tissue overdose. Calibrating these surrogates to tumor motion would provide a more accurate beam‐on trigger, although this could require daily respiratory‐correlated cone‐beam (CB) CT imaging.

**Figure 1:**
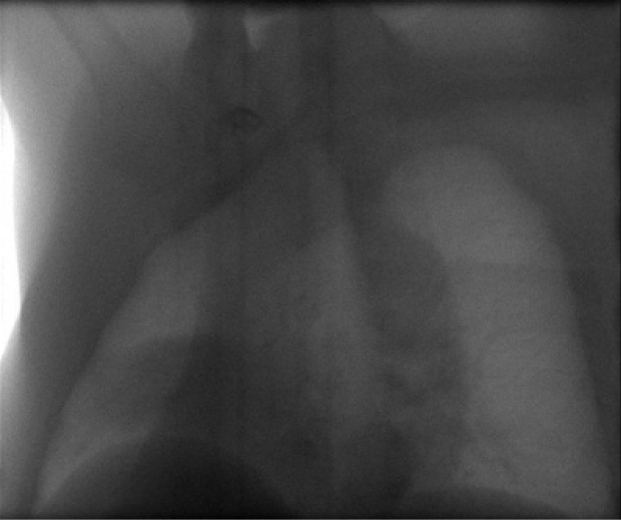
Projection image from the MVCBCT raw dataset of a NSCLC patient. The tumor is visible in the right lung just above the diaphragm.

Advances in megavoltage CBCT (MVCBCT)^(^ ^6^ ^,^ ^7^ ^)^ imaging have made it clinically possible to perform patient localization prior to each treatment by registering the treatment CT to the planning CT. This process uses the treatment beam from a linear accelerator (linac) and an electronic portal imaging device (EPID) to capture projection images as the gantry rotates. These projection images are used for reconstruction by default, or in cine mode^(8)^ where they could be exported into DICOM format for visualization and analysis. It would be useful to identify tumor positions in these projections to calibrate the strain gauge. However, the contrast in most of these projections is low and the tumor boundary is poorly defined (see Fig. 1). Tracking techniques that have been successfully applied to fluoroscopic images, such as methods based on template matching^(9)^ or optical flow,^(10)^ may not be robust enough for MVCBCT projection images.

An alternative approach may be to use the diaphragm motion since it correlates well with tumor motion for most lung cancer patients.^(11)^ In a preliminary study, we identified the ipsilateral hemidiaphragm apex (IHDA) and the superior edge of the tumor in MVCBCT projections from 27 treatment fractions of one non‐small‐cell lung cancer (NSCLC) patient. The tumor position correlates well with the IHDA position in these projection images, with an averaged coefficient of determination of 0.95 for the linear fit. Moreover, the diaphragm edge between air and tissue is clearly visible in the projection images. Previous methods have been able to detect 2D IHDA positions in MVCBCT projection images semi‐automatically, and convert them to 3D room coordinates using an interpolated ray trace algorithm.^(^ ^12^ ^,^ ^13^ ^)^ However, performing the same task for the tumor is more difficult.

One could use the MVCBCT images to quantify the relationship between tumor and diaphragm motion. Applying respiratory correlated (RC) reconstruction by retrospectively sorting all the projection data according to diaphragm position would reduce blurring significantly, enabling clinicians to identify tumor and diaphragm boundaries directly on the images. We can then derive the diaphragm‐to‐tumor motion ratio (DTMR), which is based on the tumor centroid displacement and the IHDA displacement between full exhale (FE) and full inhale (FI) CBCT images. The strain gauge signal could be calibrated for tumor motion by using the DTMR and correlating the IHDA positions with the corresponding strain gauge signal recorded for each projection image. Results based on our previous study have shown the feasibility of quantifying DTMR and tumor volume changes for a large tumor by using RC MVCBCT.^(14)^ However, RC MVCBCT has fewer projections; this causes severe view aliasing artifacts and degrades reconstructed image quality, potentially limiting its applicability to larger tumors and small displacements.

Phantom tests are needed to determine these limitations, since no ground truth can be established for patient images. At best, one can only hope to establish agreement among multiple dynamic imaging modalities such as Cine‐MR and 4DCT. Hence, in order to quantify the errors in volume and motion determination, a phantom with spherical inserts was imaged to study the feasibility of using RC MVCBCT to quantify tumor motion and tumor volumes. The actual motion of the phantom and the size of the inserts are known and serve as ground truth.

## II. MATERIALS AND METHODS

### A.1 MVCBCT

The Siemens MVision (Siemens Medical Systems, Oncology Care Systems, Concord, CA) uses an amorphous Si flat‐panel EPID with 1024×1024 detector elements, each measuring 0.4 mm×0.4 mm. The source‐to‐axis distance (SAD) is 100 cm, while the source‐to‐imager distance (SID) is 145 cm. The commercially available product uses a 6 MV treatment beam line (TBL), while a test system in our clinic uses a 4.2 MeV imaging beam line (IBL). The lower energy photons provide a better quality image for the same dose,^(^ ^15^ ^–^ ^17^ ^)^ allowing us to determine if RC MVCBCT benefits from the new beam line. For both TBL and IBL modes, the standard protocols use a 200° arc from −90° to 110°, generating one projection image per degree. We also tested a TBL protocol with a full rotation (359° arc), so that we can evaluate whether the increased number of projections improves our ability to determine tumor sizes and motion from RC MVCBCT.

We acquired 12 scans, six with the phantom at rest and six with the phantom in motion. The six scans used the three modes (200° IBL, 200° TBL, 359° TBL) at 5 and 10 MU. The phantom was also scanned at rest and in motion using 4D kVCT to compare our RC MVCBCT results against a clinical 4D system.

### A.2 Phantom

**Figure 2:**
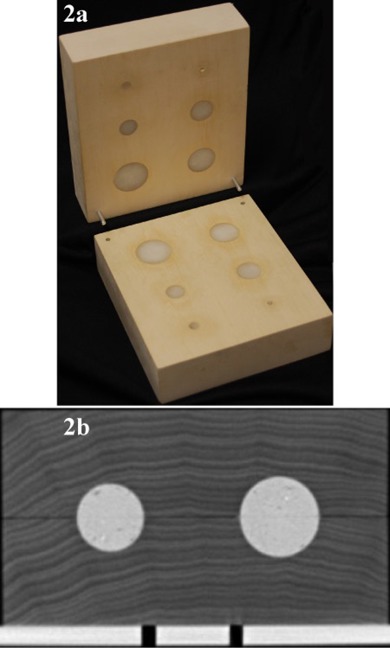
A picture of (a) the imaging phantom and (b) an axial slice through the center.

The imaging phantom consists of two symmetrical blocks of basswood, with a density of about 0.4g/cc to mimic lung tissue. Each block has six different sized hollow hemispheres measuring 3.81, 3.18, 2.54, 1.91, 0.95, and 0.48 cm in diameter, respectively. These hollow hemispheres were filled with paraffin wax, with a density of about 0.93g/cc to mimic lung tumors. The two halves were then carefully aligned to form a rectangular box embedded with six spherical pieces of paraffin wax. Figure 2 shows a picture of the phantom and a corresponding axial CT slice through the center of the phantom.

The imaging phantom is placed on a cart attached to the quasar respiratory motion (QRM) phantom (Modus Medical Devices, Inc, London, ON, Canada) to simulate respiratory motion. The QRM phantom is programmed to move only in the superior‐inferior (SI) direction, with position as a function of time, t, defined as:

(1) $$z(t)=z_{0}+A_{0}\cos^{4}(\pi (t+t_{0})/\tau )$$

The motion amplitude $A_{0}$ is 30 mm, which represents extreme tumor motion, and $z_{0}$ is the minimum position. The period, τ, is 4 s to represent typical breathing. The image acquisition started at a time $t_{0}$ after the motion phantom started moving. The designed motion and size of the spherical inserts are used as ground truth for motion and volume quantification.

### A.3 Respiratory signal detection

To detect the respiratory signal in the MVCBCT projection images, we developed an in‐house platform using Microsoft Visual Basic 2005 (Microsoft Corp. Redmond, WA). An algorithm based on the interpolated ray trace method^(13)^ confines the respiratory motion of an object into a bounded rectangle for each projection image. It requires a user to manually identify a tracking point (such as the apex of a hemidiaphragm for the clinical case) in two FE frames and two FI frames. This step typically takes 20 seconds for the diaphragm apex. Applying trigonometric relationships among these four points allows us to determine a smaller region of interest where the motion is contained. Dynamic Hough transforms based on parabolas are used to create cost functions, while dynamic programming is used to minimize the cost functions to find the trajectory of the diaphragm. (The complete details are given in References 12 though 14.) In this study, we used the center of the largest spherical insert as the tracking point. Using an algorithm based on a Hough transform for circle detection,^(18)^ we are able to detect the largest spherical insert within the rectangular region. Parameter tuning and manual correction is performed when a detection error occurs. Since the errors are limited to a few frames, this correction step takes less than 20 seconds. (Future work involves using robust correlation to predict the required correction and eliminate the manual correction step.) Having identified the position of the center of the largest insert in each projection, the 3D position as a function of time can be approximated by a sequence of trigonometric relationships, as described for the interpolated ray trace algorithm.^(13)^

### A.4 Respiratory correlated reconstruction

The craniocaudal motion of the center of the spherical insert is automatically rescaled into a relative motion range from 0 to 100, which correspond to the most superior and the most inferior positions, respectively. In previous lung cancer patient studies, we applied the rescaling to the motion of the apex of the hemidiaphragm below the tumor.^(12)^ 3D images at FE (0%) and FI (100%) are sufficient to quantify tumor displacement between full exhale and inhale states. Projections are sorted to these two respiratory states with a fixed amplitude interval. The size of the allowed amplitude interval is a compromise between view aliasing artifacts (the reduction of which requires more projections) and residual motion (the reduction of which requires fewer projections). The interval is set at 10%, which corresponds to 3.0 mm in our study. Hence, the FE image is reconstructed from projections with sphere positions of approximately 0 to 3 mm from full exhale position. For the FI image, the selected projections have sphere positions of approximately 27 to 30 mm inferior to the full exhale position. For a CBCT scan that acquires 200 projections of a phantom that moves according to Eq. (1), about 70 projections are included in the FE phase, while about 30 belong to the FI phase.

Image reconstruction was performed using the Feldkamp, Davis, Kress (FDK) method.^(19)^ For the MVCBCT imaging of a static phantom, a clinical FDK reconstruction system is used. The dimension of the 3D volumetric image is 256×256×274 (274 is in the craniocaudal direction), with a voxel spacing of 1.0 mm. For imaging of the moving phantom, an offline FDK algorithm is developed for the RC reconstruction. (The offline application uses the same algorithm as the online version, but it is used to reduce demand for the clinical imaging workstation that performs the online reconstruction.) The sorted projections are reconstructed into an image of 256×256×256, with a voxel spacing of 1.071 mm. The dimension of each 3D image for the 4DCT scan is 512×512×274 (274 in the craniocaudal direction), with a transverse slice thickness of 1.0 mm.

### A.5 Quantification of motion and volume

All the 3D MVCBCT and kVCT volumetric images were stored in DICOM format and imported into the Philips Pinnacle (Philips Medical Systems, Andover, MA) treatment planning system. All spherical inserts in all the images were contoured. The volume and center of the contoured regions of interest were derived using Pinnacle's measurement tools. The displacements between volumes in the FE and FI images were computed from the difference of the centroid positions.

Previous studies have demonstrated that the display window center (WC) and window width (WW) significantly influence the apparent size of an object in CT imaging.^(^ ^20^ ^,^ ^21^ ^)^ It was found that the WC should be half of the attenuation differences between the object and the background in order to yield the correct size. In this study, we set the WC at half of the attenuation difference between the spherical insert and the basswood frame. The WW is set as the attenuation difference.

## III. RESULTS & DISCUSSION

### A.1 Image quality

**Figure 3:**
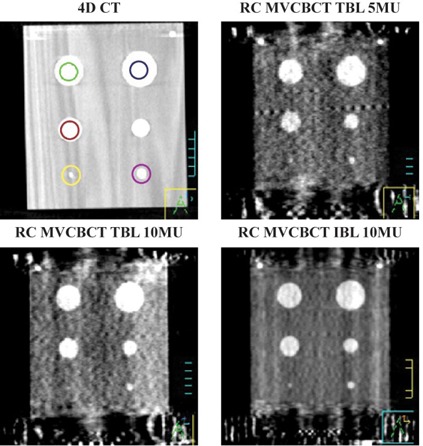
One coronal slice of the phantom imaged using kVCT (top‐left), RC MVCBCT with 5 MU TBL (top‐right), RC MVCBCT with 10 MU TBL (bottom‐left), and RC MVCBCT with 10 MU IBL (bottom‐right).

**Figure 4:**
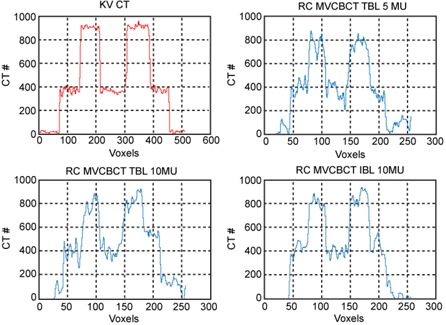
Imaging profile of the two largest inserts in FE phase images using kVCT (top‐left), RC MVCBCT with 5 MU TBL (top‐right), RC MVCBCT with 10 MU TBL (bottom‐left), and RC MVCBCT with 10 MU IBL (bottom‐right)

Figure 3 shows one coronal slice of the FE phase for kVCT and RC MVCBCT imaging for different protocols. Figure 4 compares the attenuation profiles of those images for the largest two spherical inserts. For the kVCT, the image intensity is distributed uniformly within each spherical insert and the CT number represents the material density well. For the other three RC MVCBCT images, the image intensity is no longer uniformly distributed, as noise occurs in both wax and basswood regions. There is some difference between the CT number in the RC MVCBCT and the CT number that corresponds to the actual density of the material. As expected, the uniformity within the sphere improves as the dose is increased from 5 to 10 MU (from upper right to lower left panel), and as one goes to a softer energy spectrum (from lower left, TBL, to lower right, IBL). The smallest insert is identifiable in images reconstructed using IBL or images reconstructed from 359 projections. Using a wider range of projections and IBL improves the imaging quality.

### B.2 Volume determination

**Figure 5:**
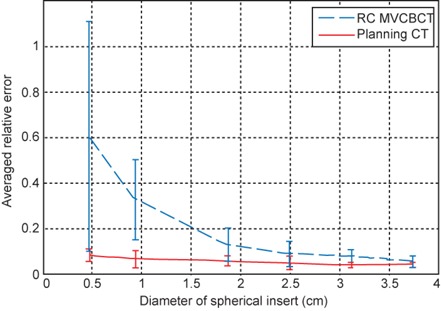
Relative error in volume as a function of the phantom insert diameter.

**Figure 6:**
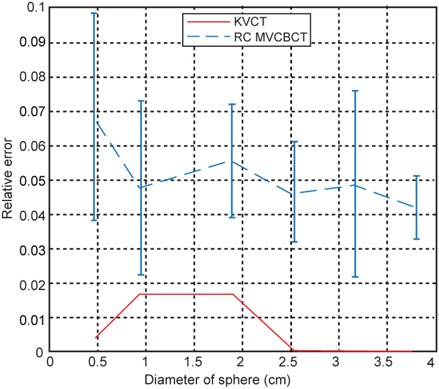
Relative error in displacement as a function of the phantom insert diameter.

We use the relative error to measure the accuracy of volume determination. The relative error is defined as the normalized difference with the nominal designed value:

(2) $$\text{Relative}\;\text{Error}=\left|V_{actual}-V_{designed}\right|/V_{designed}$$

where $V_{\text{actual}}$ is the volume measured from the contours and $V_{\text{designed}}$ is the nominal designed volume. Figure 5 compares the average and standard deviation of the relative error in volume for all the kVCT images and RC MVCBCT images. For the planning CT, the error is within 10% for all the inserts of different sizes. For RC MVCBCT, an inverse relationship between object size and relative error is present. Image degradation due to view aliasing artifacts and residual motion only affects the apparent size of the border region for large objects, but may affect the small object entirely. The image pixel dimension also affects smaller objects, since it is a larger fraction of the object's diameter. The residual motion of 3 mm also has a greater effect on smaller objects. The average error of the four larger inserts is about 10%, but errors increase significantly when the object diameter is less than 1 cm, indicating that volume measurement in RC MVCBCT is not suitable for small objects. For larger tumors, we have observed tumor volume reduction through a course of treatment using methods similar to what is described here (see Fig. 6 in Reference 14), so RC MVCBCT could provide tumor response assessments for tumors larger than 2 cm in diameter, as verified by 4D kVCT imaging. ^(14)^ However, the case study was for a regular breather who provided a sufficient distribution of uniformly spaced projections for RC MVCBCT. Irregular breathers will most likely have fewer projections with irregular spacing and could induce more artifacts. Further studies with irregular breathing patterns programmed into the phantom would be needed to determine the limitations on these situations.

We further reclassified the results based on different imaging parameters to study their influence on the accuracy of volume determination. Tables 1 to 4 show the averages and standard deviations of relative errors belonging to different subsets of imaging parameters, including different respiratory states (Table 1), number of projections (Table 2), imaging dose (Table 3) and source energy (Table 4).

**Table 1:** Average and standard deviation of relative volume error for FE and FI phases.

*Sphere Diameter (cm)*	*Static*	*FE Phase*	*FI Phase*
3.81	3.02%±1.53%	5.81%±2.03%	4.74%±3.12%
3.18	2.60%±2.28%	8.42%±2.95%	7.08%±3.03%
2.54	3.08%±2.51%	10.6%±5.20%	6.43%±5.26%
1.91	7.95%±10.3%	15.8%±5.65%	9.52%±8.11%
0.95	8.11%±6.73%	33.6%±20.3%	31.4%±16.4%
0.48	16.7%±10.1%	27.6%±19.9%	100%±48.3%

**Table 2:** Average and standard deviation of relative volume error for different arcs.

*Sphere Diameter (cm)*	*200°*	*359°*
3.81	2.84%±1.70%	5.91%±3.40%
3.18	6.33%±3.81%	5.12%±3.87%
2.54	8.09%±6.43%	3.62%±2.14%
1.91	11.8%±9.47%	6.58%±4.38%
0.95	21.3%±18.8%	19.3%±18.1%
0.48	55.8%±48.8%	36.2%±25.7%

**Table 3:** Average and standard deviation of relative volume error for 5 and 10 MU.

*Sphere Diameter (cm)*	*5 MU*	*10 MU*
3.81	3.66%±2.06%	5.38%±2.69%
3.18	4.52%±3.15%	7.54%±3.64%
2.54	5.29%±4.33%	8.08%±6.01%
1.91	8.20%±5.91%	14.0%±10.0%
0.95	23.9%±18.0%	24.7%±20.7%
0.48	42.1%±52.8%	47.7%±41.8%

**Table 4:** Average and standard deviation of relative volume error for different energies.

*Sphere Diameter (cm)*	*kVCT*	*IBL MVCBCT*	*TBL MVCBCT*
3.81	4.02%±1.04%	4.81%±0.85%	4.37%±3.02%
3.18	3.80%±1.10%	6.65%±3.79%	5.72%±3.71%
2.54	4.69%±2.94%	8.36%±5.64%	5.86%±5.13%
1.91	5.66%±2.28%	14.8%±9.76%	9.21%±7.55%
0.95	6.37%±3.89%	32.5%±20.1%	20.3%±17.6%
0.4	7.98%±2.76%	45.0%±62.8%	45.1%±37.3%

In Table 1, the relative error of static objects using standard MVCBCT is significantly smaller than that for FE or FI images using RC reconstruction. The error using static object MVCBCT data is even comparable to that of the kVCT for the four larger inserts. This is, in part, a consequence of using fewer projections, as can be seen as well in Table 2 where the relative errors for a complete rotation (359°) are lower than those for a 200° arc. However, one would expect that since the FE phase has more projections (70) than the FI phase (40), the FE phase should have better accuracy. This seems to be true only for the smallest sphere, while for the larger spheres, the FI phase is slightly better. This could be a consequence of the actual residual motion in the reconstruction. Although the projection sorting algorithm used a 3 mm window, the actual residual motion could be slightly smaller for the FI phase than for the FE phase. In fact, for the FI phase, typically only two projections were selected per respiratory cycle. With fewer projections in the FI phase, the likelihood of spanning the entire 3 mm window is lower.

It is very likely that more projections available in a wider range of angles for each phase may reduce the view aliasing artifacts for tumors, although the effect of these artifacts on volume determination for spherical objects seems to be less of an issue than residual motion. However, using a 359° rotation increases the image acquisition time. This presents a compromise between reducing setup time and finding a more accurate protocol.

Tables 3and 4 present counterintuitive results. One would expect higher imaging doses to produce better images and, hence, improved volume determination. Similarly, one would expect softer energies to yield lower volume errors due to improved image quality. Figure 3 shows how image quality improves according to this expected pattern. Tables 3 and 4, however, show the opposite trend. The differences, however, are within the standard deviations. Within experimental error, they essentially produce the same result. It is possible that the amount of residual motion varies quite a bit due to the random starting phase for image acquisition, and this is just enough to affect the results.

### C.3 Motion quantification

Similar to volume determination, we also use relative error to present the normalized accuracy of motion quantification, expressed as:

(3) $$\text{Relative}\;\text{Error}=\left|M_{actual}-M_{designed}\right|/M_{designed}$$

where *M* represents the displacement of the centroid between FE and FI respiratory states and the subscripts are consistent with those in Eq. (2). Figure 6 shows the average and standard deviation of the relative error of the motion of the six spherical inserts when using kVCT and RC MVCBCT. It should be noted that there is only one kVCT scan of a moving object. The measured displacement is very accurate for this kVCT scan. The three largest inserts have exactly the same motion measurement as the nominal designed value of 30 mm. The error for the smallest of the three inserts is within 2%. For RC MVCBCT, the errors for the five largest inserts are all about 5%. The error is slightly larger for the smallest insert at 6.8%. All the relative error of motion is within 10%, which correlates well with the 10% amplitude interval in amplitude‐based projection sorting.

Since the difference of motion quantification between different inserts is small, we present the average and standard deviation of the relative error in Table 5 by summarizing all the inserts belonging to the same type of RC MVCBCT scan. The nomenclature for the various imaging parameters is consistent with that of the previous section. The difference in relative error is very small (0.3%) between different imaging parameters, indicating that motion quantification is comparably more robust and insensitive to variation in manual contouring than volume determination. It is feasible to quantify tumor motion amplitudes between FE and FI respiratory states by using RC MVCBCT, even for objects with a diameter of about 0.5 cm. It should be noted that typical tumor motion amplitudes range from 1.0 cm to 2.5 cm, which is smaller than the phantom motion in this study. This gives us confidence to extend the practice of evaluating motion between the FE and FI respiratory states of 4DCT data to cases of RC MVCBCT taken immediately prior to treatment, to determine if the maximum motion is consistent with the one determined at the time of treatment planning.

**Table 5:** Average and standard deviation of relative error of motion using kVCT and RC MVCBCT.

*Imaging Parameter*	*Relative Error*
*kVCT*	0.61%±0.83%
All RC MVCBCT	5.06%±2.14%
IBL	5.00%±2.07%
TBL	5.09%±2.22%
5MU	4.90%±2.21%
10MU	5.20%±2.13%
TBL 200	4.94%±2.41%
TBL 359	5.22%±2.13%

### D.3 Application to patients

Because the study used spheres, they may be less susceptible to view aliasing artifacts. Volume determination for tumors may be less accurate than what is noted here, but these studies at least establish a lower limit on tumor sizes that can be evaluated with RC MVCBCT. For motion assessment, however, since the centroid of the tumor is used, it will be less sensitive to the identification of the tumor edges. This could explain why the accuracy for motion assessment is more robust. This could carry over into patients as well, and manual identification of the tumor in projection images for a test patient^(^ ^12^ ^,^ ^14^ ^)^ showed that the tumor displacements between full inhale and exhale, averaged over all imaged respiratory cycles, was within 3 to 5 mm of the value determined from RC MVCBCT.

When one determines volumes and motion within a patient, they need to know whether their imaging methods are causing errors. Using spheres allows us to reduce the possible errors coming from user variability in contouring, and instead allows us to determine possible errors that come from the reconstruction of fewer projections than what one would normally expect. Our previous feasibility study for a patient^(14)^ had to rely on comparison of the RC MVCBCT results against the results from the 4D planning CT. While volumes can be compared for MVCBCT and 4DCT images taken on the same day, one can not compare the amplitude of motion from full exhale to full inhale since the respiratory motion for the two separate imaging sessions may be different. This is the best one can do for patient studies, since the true motion and volume of the tumor can not be established; even the 4D planning CT will contain residual motion and artifacts, and its usefulness in serving as ground truth is subject to these errors. The results in our phantom studies provide some lower bound on errors, since the error may be greater due to inaccuracies in contouring nonspherical objects and reconstruction errors arising from irregular breathing patterns. More work is needed to verify our results using phantoms with realistically‐shaped tumors.

With respect to imaging the IHDA in order to sort projections, one must carefully choose the CBCT isocenter. For very lateral lesions on the Siemens MVision system (27.4 cm FOV at isocenter), or for large patients, we often have to shift the patient about 5 cm medially to avoid collisions. This has not caused problems for viewing the ipsilateral hemidiaphragm apex in all the protocols that we have used clinically. On other CBCT systems, a half‐fan option is available, and this may present problems for finding the IHDA. To use the techniques described here for patients, one must carefully understand the limitations of their imaging devices and protocols. A protocol using an angular range that keeps the IHDA in view would be preferable. The methods presented here can also be applied to test these alternative protocols.

## IV. CONCLUSIONS

In this study, we have evaluated the feasibility of using RC MVCBCT to quantify object motion and size. The primary source of object boundary detection errors is the reconstruction error induced by missing projections. Better accuracy can be achieved for volume determination when the object is sufficiently large (a minimum diameter of 2 cm). For larger tumors, response assessment in terms of volume reduction is feasible for regular breathers, at least until the tumor shrinks down to 2 cm, where a 4D kVCT would be needed for volume determination. Motion measurement results, on the other hand, are more robust. The relative error is within 10% for even the smallest object, and it is independent of energy, dose and protocol. This would allow us to relate diaphragm motion to tumor motion which, in turn, could be used for calibrating surrogates of tumor motion (since we can track the diaphragm in the projections), even for the smallest lung tumors that are clinically encountered in radiotherapy. The phantom studies in this work can serve as a quality assurance method for any type of respiratory‐correlated imaging, since they provide ground truth for size and motion, which cannot be unequivocally established with patient images.
